# A New Species of Dentist—Do We Want It?

**Published:** 1920-03

**Authors:** Thomas J. Barrett

**Affiliations:** Worcester, Mass.


### A NEW SPECIES OF DENTIST—DO WE WANT IT?

BY THOMAS J. BARRETT, D.D.S., A.M., WORCESTER, MASS.

When I received your kind invitation to come here and speak upon a subject which I considered of great importance to us, I was somewhat in doubt whether my enthusiasm for professional ideals did not outrun my ability to present to you in the manner which I view it the evil consequences that lie in the wake of any attempt to commercialize professional ideals. I feel, however, that I am no stranger to Pennsylvania, because it was within the confines of your state that I was taught enough dental lore to enable me to pass the examinations that entitled me to my degree. Nor can I efface from my memory the high ideals and standards that the learned professors of that day sought to impress indelibly upon our young and untrained minds. I shall, however, attempt in my humble way to present to you some of the reasons why I am opposed to legislation which, as I view it, will tend to lower the high standard which we have attained, and which I hope will not only be maintained, but raised to a higher grade.

The original characterization of this new species was dental nurse; latterly it is dental hygienist. Under either designation her advent is one of false pretense. The various statutes that I am at all familiar with outline her legal existence as one authorized to clean the exposed surfaces of the teeth. Any one at all familiar with the definition of the word nurse, or nursing, would not apply either to that work; nor would one familiar with the word hygiene coin the word hygienist, and thus apply it.

Under her present designation, hygienist, one not fully informed as to her legal status and educational limitations would likely assume that this titled person was a highly-trained specialist in dental and oral hygiene, an assumption that is far from the facts and truth, as I have observed them, and as I have examined her work.

Permit me at the outset to give you a brief history of the development and evolution of this new species. The state of Connecticut, I believe, has the honor (?) of being the first to recognize and legalize this new creation in dentistry. Until within recent years a registered dentist in that state was permitted under the law to employ non-registered assistants under his supervision or direction. That allowed dentists employing secretaries, typewriters, stenographers, or plain office girls to spend their leisure time, profitably to the office, in cleaning teeth, and many of them did so spend their time. The most ardent supporter of the so-called dental nurse in that state, openly and with much outward show of sincerity and truth, declared at public hearings in Massachusetts that these women, thus trained on the typewriter or office index and a course in observation at his chair and clinic, could and did clean teeth better than any dental college graduate he ever knew.

The first of this new species I ever met came from the state of the historic wooden nutmeg. The germ spread quickly, however, even to the "wealthy, cultured and refined'' section of my own state, known as the Back Bay in Boston. Several of the leading men of that location saw at once the possibilities of profit, with or without charity, in this new idea, and decided it was worth developing. They invited me to a conference on a proposed bill containing the new idea that was to be presented to the legislature for enactment, away back in 1906. This was the first attempt to legalize a dental nurse in Massachusetts.

The bill presented for consideration provided that any girl, eighteen years of age or over, of good moral character, on payment of a fee of $5, could take an examination before the state board of dental examiners, and if found qualified should be licensed to clean teeth, change dressings in teeth, and otherwise assist in the office of her employer. The changing of dressings in diseased teeth by a person trained as I have stated above was too much for me to indorse, so without waiting to consider the less harmful proposal of cleaning teeth, I opposed it, and had no further discussion with them on that bill. Such credentials of fitness to permit persons to operate in the mouth, I did not think met the professional requirements we as a profession should exact, even though as a member of the board I would have opportunity to examine the candidates.

The requirements for the examination, as I have stated above, consisted of the $5 fee, a good moral character and the candidate must be eighteen years of age. And yet the advocates of this original bill in Massachusetts to legalize the dental nurse organized committees, employed attorneys and legislative agents, and went about the work of securing legislation favorable to their desires in the most systematic manner that their ingenuity could suggest, and observing lobbyists and politicians could advise and direct. But they did not succeed in passing the bill.

All this committee work, this legislative activity, circular letters by the score, personal appeals, and I do not know what else, were employed, save that every known method to secure legislation was used to aid and promote this "nurse" bill. The same methods were employed and vigorously used, year after year, by this band of "charity workers." Their strongest argument was that it was all for and in behalf of the poor people and preventive dentistry. It was pointed out by them that the future welfare and health of the people of the great state of Massachusetts, yes, I might add the health and stability of generations yet unborn, depended on legalizing the dental nurse for charity work; but mark you, she must also be permitted in private practice under the direct supervision and direction of the registered dentist. That clause, the joker in the bill, was to work as an even offset to the charity clause in the bill, so that the charity would also come home in a substantial way to the workers for the bill.

During the seven or eight years of active legislative work I did against this measure it is safe to say that twenty-five, yes thirty-five, different bills were presented, and of every one presented not one appeared without the private practice privilege. I challenge this sort of charity and this sort of interest in public health. I repudiate it. It is rank hypocrisy. I did openly challenge it, to test its sincerity, by saying, "I will withdraw every word of opposition to the dental nurse I have ever uttered, if you people favoring this bill will withdraw from your bill all that relates to the nurse in private practice. I will accept all that relates to the nurse in charitable institutions, public schools, dispensaries, clinics, etc." The answer came back from the active leader, "My patients are as much entitled to the benefits of this work as are the poor." I submitted my case on that answer as to motive. The nurse in private practice is and was the one thing sought, as you men of Pennsylvania may soon find out, and I urge you to stand guard.

In Massachusetts practically every active supporter of the legislation for the dental hygienist subscribed liberally to the expense incurred in promoting the drive for the bill and fighting the opposition to this so-called public health and charity measure. Today they are nearly all receiving in return full payment, plus liberal interest on these contributions through the earning power of their associated dental nurse.

Does any one believe that you could train and equip at the present-day low standard of educational requirements a sufficient number of dental hygienists to give personal care to the teeth of all children of this great state, to say nothing of national needs, and all of this to be at public expense? That is a thought "made in Germany," and much resembles paternalism and German "kultur," and not at all likely in this or any other state.

Every hygienist graduated from the Forsyth Infirmary and ninety-eight per cent. of all from Eastman, if I am correctly informed, have found their way into private practice, not one to the field of charity. It is time to push gently aside this cloak of charity that has been the foundation stone of all the legislation thus far granted to this new species of half-trained oral operators, and to tell the facts just as they are. It is much easier and the prospects much brighter for success to approach law-making bodies with a proposition that has charity as its object and foreword than to seek the same ends through frank admission of private gain.

The most prominent propagandist in New England for the dental hygienist, being cross-examined by a member of the Committee on Public Health of the Massachusetts Legislature, stated that he employed two nurses in his office. One he paid twenty-five dollars per week, and charged three dollars per hour for her services. She worked seven hours a day, five days of the week, and four hours one day; he also stated that her time was fully occupied.

Thirty-nine hours at three dollars per hour gives you one hundred and seventeen dollars, and from that subtract twenty-five dollars and you have the income from one nurse, with the returns from the other not yet reported, as well as a good idea of the charity that guides the movement. It always begins at home, even though you dearly love the common people, and charity is your public work.

The world at the present time is seething with false propaganda, and I am opposed to this particular propaganda, either under the subterfuge of deep interest in public health or charity, and wish any one of the active advocates of the nurse to point out any place where it is in operation. If the poor are to have free dental services of any description, they should have the very best or none at all. They will be far better off with none than with that rendered by the inferior and unskilled.

In communities that can in any way, by public or private funds, afford it, dental clinics should be opened, equipped with the best and most modern appliances for their safety and well-being, and the services of the best trained men obtained by paying such of them for their skill and time as will be found necessary.

### PREVENTIVE DENTISTRY

Much has been said of late on this subject, and many arguments favoring the dental nurse or hygienist contain this phrase—it is catchy and sounds good—as the foundation stone of their support of the nurse propaganda. The propagandist is forceful in outlining the advantages that oral hygiene has in relation to tooth decay, and he goes so far along this line that he takes away from the skilled, educated and experienced dentist everything but the mechanical tools, and turns all else over to the hygienist. She is the one to teach the people preventive dentistry, and according to some eminent writers is the only one who understands and has the patience to practice it. The real busy dentist has not time to devote to it; he frankly tells you so, in the words of the nurse advocates. The dentist is the mechanic, the tradesman; he builds bridges and repairs teeth.

If the diseases of the teeth and mouth are preventable, the profession should put forth its best efforts to educate and enlighten the people on the simplest and best methods to employ to retain and obtain healthy mouths and teeth. Not tell them that their only salvation depends on patronizing the nurse in private practice or in public institutions.

Ridiculous arguments, and yet used by writers and oral advocates of the nurse! The research department of the National Dental Association, busily and studiously engaged day after day in an endeavor to develop information for guidance and enlightenment of the profession and for the better health of our people, can abandon its efforts. Preventive dentistry in its entirety lies only in the direction of the hygienist, if the nurse advocates are to be believed.

Many of these propagandists openly and loudly assert that the dental nurse is far superior as a technician in prophylaxis to the college-trained dentist. Such a statement is not only absurd, but is insulting to the members of the profession. I have examined both, and I resent with all possible force such a statement.

I am told that a recent ordinance passed in New York provides for a dental clinic. Appropriations for its maintenance include salaries for a registered dentist in charge at the rate of $1,020 per year, and a hygienist at $960 per year. The educated dentist, with three years of college work, is rated $5 per month higher in the wage scale than the hygienist after eight months of training. Such is professional ethics when the active leaders get a fad and a desire for a profitable hobby.

We as a profession are fully agreed that preventive dentistry and improved public health lie in the direction of oral cleanliness. Then let us as dentists assume the responsibility for the proper enlightenment and thorough presentation to the public of the best methods for them to use. This is our work, and its development our duty. Let us not turn it over to the hygienist, and say we are too busy to give this great work that will promote and maintain health the attention it deserves. Let us not say by word or by attitude, dentists only repair teeth and supply substitutes and that the science of saving teeth and improving health is in the hands of the dental nurse. Heaven forbid!

I am opposed to any one being allowed to operate in the mouth under a lower standard of educational requirements than is at present demanded of the dental student. The three years' course for dental students proved insufficient to meet the requirements. The public and the profession demanded a better product; we now require a minimum of four years. Are we going to open wide the doors of opportunity to a species of dental adventurers to operate in the mouth after seven or eight months' training? I hope the answer from the great state of Pennsylvania will be a vigorous "No." Do not let down the bars for such camouflage.

I have been a state examiner for twenty-five years and have examined and re-examined fourteen thousand dentists, and feel that I ought to be well acquainted with and possessed of as much knowledge of the average dentist's ability, his ethics and character, as any man in the profession, and know how this new species with from seven to eight months' training will fill a long-felt want in some offices.

I believe I know some, and think you may know many offices where the hygienist will always be permitted to earn her salary, and as much of the office expense as the dentist under whose direction she may be operating at the time will consider prudent and profitable. It is not at all likely that in such places the legal limitations restricting her work under all conditions will prevail. It is contrary to all known examples of human frailty.

In the most offhand manner, you are told by editorial writers in some of our dental literature that the police powers of the state will take care of all violators of the law. Any one with any experience in attempting to protect the public from the ignorant and incompetent practitioner knows that the laws we now have to safeguard them are unable to reach them except in the most flagrant cases.

How would it be possible to reach the registered hygienist licensed to operate in the mouth; licensed to have at her command and ready for use every instrument and appliance known to dentistry? Who could discover violations under such conditions? No one save the patient in the chair could convict a licensed hygienist disposed to go beyond her legal limitations. I feel sure you can not hold her under the direction of the dentist for any length of time, any more than I believe you can restrict her to cleaning teeth for a life's work. She will overcome the first barrier through legislation, and the second through a desire to aid the office in her leisure or some other equally strong motive.

COMPARISON OF THE DENTAL NURSE TO THE MEDICAL NURSE

Many comparisons are made by the propagandists with the medical nurse; there is nothing in common between them. That is so evident to the medical nurses that their organization was represented at the hearings on the bills in Massachusetts, and filed strong protest before the Committee on Public Health in Massachusetts against the use of the word nurse as applied to this new species. That protest forced a change of name, and the word hygienist was coined to fit.

It has been urged in argument, and may be again, that the precedent established by the medical profession might with success be followed by us. But the cases are not analogous. The medical nurse generally performs most of her services in the home and in the absence of the physician, or at the hospital in the temporary presence of the physician. The surgical nurse assists the surgeon, but never attempts to perform even the most trivial operation. And, furthermore, medical nurses are equipped with a three years' training course. The hygienist, on the other hand, is at most a one-year product, and unlike the medical or surgical nurse, does her own operating, provided she is in the constructive presence of her employer. The reasons for her existence are not founded in theory or practice upon the same grounds as the nurses who assist the medical profession. Even the medical nurses protest against the use of the word nurse in connection with this species in dentistry, because they are proud of their work and resent the importation of an inferior article.

The medical nurse is the finished product of a thorough educational system, with three years of hard and thorough training divided between theory and practice, day and night. She is not any short-cut, suddenly developed specialist.

We have laws legalizing dental nurses in from four to six states at the present time, and in every one of the laws enacted there is a binding provision to the effect that this nurse or hygienist shall work under the direction and supervision of a registered dentist, absolutely. In other words, those who framed and caused to be enacted these various laws saw to it that the hygienist or nurse would be tied by law to them, and could operate or practice only under their direction and in their office.

Now, I submit to you that this shows one of two things: either her incompetence is acknowledged or a revenue from her earnings is desired. When this nurse or hygienist is legislated into being and is examined by the state board—under the law creating her or him—and meets the requirements of the law and is duly registered, she then should be entitled to practice her calling as a nurse or hygienist without further restrictions or restraint. She is either fit to practice or she is unfit. If she is fit, why does she need direction? If she is unfit, why should she be registered, or allowed to practice at all? If that is not good logic, I do not know what is. She either meets the requirements of the state law as to her fitness or she fails. If she meets them, she does not need your guidance, and she should be allowed to go forth under her own direction. New York is the only state where the law says "her." It says "him" in Massachusetts, and I can see within a short time she or he will not ask for your approval nor submit to your direction or supervision, but will gain liberty by changing and amending the laws that bind, and go it alone as specialists in dental hygiene.

To those who object to her being granted liberty and freedom in the pursuit of a living, unhampered and uncontrolled, and fortified under a license granted by the state, the pertinent question may properly be asked: Is your objection to her freedom in the interest of the dentist who profits by her legal custody, or in the interest of the public, whom you say she is competent to serve? Which may I ask? When this day of liberty and freedom comes to the hygienist—and it is not far distant in some states, if I am correctly informed—then we shall have the tooth-cleaning department added to the manicuring, hair-dressing and chiropody facilities of the up-to-date department stores and hotel barber shops.

The dental nurse was originally intended for the busy practitioner with a practice among those who would appreciate the benefits of polished teeth and a gum massage. I have had the pleasure of inspecting the mouths of many of these patients who have had the price, and paid it, for the benefits of this exclusive treatment. I have lived long enough to know that there is not and never can be a sufficiency of the good things of this life to go around on a basis of equality, and consequently the full enjoyment of this exclusive treatment will always be limited to a small minority.

If you will turn your direct gaze and attention on the advocates of the nurse in private practice, instead of on the theories they profess and ignore; if you will ask to have pointed out what charity and public interest they have ever shown and directed in this great cause, you will find that their interest in preventive dentistry does not extend much beyond their own office. It is on account of this interest that they attack those who oppose them, and start a network of useless controversy about theories and results here and there, which even if they could not be conclusively proved to be impracticable, would still not justify the nurse in private practice. It is quite natural that poor, suffering humanity struggling for dental relief and improvement should be blinded by this false light and hope. Let the profession speak out, and through its societies, state and national, put an end to this false propaganda.

There are some well-intentioned persons attracted by theories being spread abroad, who without realizing that they are intended to keep the minds of men from consideration of the real issue, see only that there is a possible means of benefiting the general health of the children, and that it might do good. Such people only add the weight of their influence to the self-seeker.

### THE PROPER MEANS OF MEETING THE NEED FOR ORAL HYGIENE

What is our duty, then, as a great profession? Is it to indorse this movement for the nurse in private practice, or to devise ways and means to meet the national needs in an effort to overcome diseases of the mouth in the millions of our people? The vital importance of this condition concerns the whole dental and medical professions, and the departments of health, hygiene and education in every state, city and hamlet in this country. To meet this situation with any hope of improving it, we must do it by liberal and democratic education, open to all.

There is no question about the cleverness of the nurse propaganda, in that it has set the dentists and some civic workers to talking about something that has no direct bearing on the situation that confronts the dentists of this country. It throws dust in the eyes under cover of so-called theories that could never be worked out, and hides the real issue.

Twenty-five million school children alone in this country. Are we going to provide dental nurses for all of them, and on an eight months' basis of education, and then operate the clinics at public expense? Well, we shall have quite an imposing array of nurses on the payroll doing preventive dentistry, and with woman suffrage we can see the present type of dentist looking for a safe place to sit down.

The New York authorities in their recent ordinance rated the registered dentist only $5 per month higher than the hygienist, and no doubt they will have many applicants for the place. Why not take the young dentists, at this cut rate in professional service, the recent graduates, train them further if found necessary to meet the exacting requirements of the nurse advocates, so that they may specialize in oral hygiene? Let them do this work in the offices of the busy dentists, the dentist who must have the hygienist to keep apace with his practice, the demands of his patients, and the desire of his pocket.

If the dentist pleads that he is unable to devote his time to such important work as preventive dentistry, then the profession will be forced to do as tradesmen, who under the pressure of competition and in order to maintain their necessary income, cheapen their products and increase their marketable output.

Here you have men who after this year must give four long years to acquiring sufficient knowledge to be deemed safe to operate in the mouth. Will you create a new species, with an eight months' course in training, and allow them the use of every instrument from the scaler and the scalpel to the electric engine and the X-ray? Will you allow them to operate in the mouth on a few months' training with the entire dental outfit at hand, and expect that conscience alone will keep them scrubbing teeth and doing nothing else?

I believe there is a place for a properly trained, educated hygienist or dental nurse. There is a field for this work in the public schools, in charitable institutions, in hospitals, dispensaries, out-clinics and the like, but, gentlemen, there is no place for her in private or public practice on a lowered educational standard, absolutely none.

The great accomplishments of American womanhood during the war have added to their creditable record, and woman has made her place on a parity with man. It is not necessary to curtail her limitations nor to abridge her rights. Woman did not set the standard for the education and training of the hygienists; as such she did not exist. She has been created, called into legal existence in some states by some of the men of the dental profession, and they set the standard, and set it so low that it discredits her. I know of no one who objects to her coming if she attain the standard of education and training that the environment she is called on to enter requires and demands.

We have eminent medical authority warning their patients and the public as well against the methods we now use in root-canal fillings, and against all crown work on devitalized teeth; against all bridgework where abutments are devitalized teeth; against all fillings in devitalized teeth.

On the one side, we have medical men openly advising the public against our methods of practice as injurious to health, and the revelations of the X-ray justify to an extent this advice. On the other hand, you have certain men who openly acknowledge their time too valuable to practice the best methods known at the present time of preventing decay and oral diseases, yet are unable to meet the demands of their patients or the public, either in the treatment of decay and its attendant results or pyorrhea and its systemic effects. And these same men offer as a solution of this grave situation the dental hygienist, with from four months to a one-year course of training and education.

Whither are we drifting as a great profession? We are raising educational requirements to four years at one entrance to the profession, and establishing another entrance where one year or less will suffice, and permit the legal use of every instrument and appliance that the four-year product can use. Their only restrictions against doing every operation and every treatment of the four-year product are the honesty of their employer and their own conscience.

Now, then, I believe we are going too fast and that but a small per cent. of the profession favor the hygienist in private practice, and that much harm will follow in her trail. I believe the profession in general and this organization in particular should go on record along conservative and sane lines that have been advocated in years past.

Oral hygiene as well as general hygiene of the body should originate at home; should be practiced there. Here is the field for dental education along the lines of oral cleanliness. It is the solemn duty of the dental profession to arouse the public to the importance of clean mouths. It should be made a part of the curriculum of every school in this country; children will then receive both home and school instruction and reminders, rather than an occasional scrub up at some public tooth-cleaning station in charge of the nurse.

I believe the responsibility for enlightening the public is squarely upon the dentist and the dental organizations of this country, not upon the so-called nurse.

I further believe:

(1) That the first duty of the dental profession is to secure the active co-operation of the medical profession in the crusade for clean mouths as an absolute necessity for health.

(2) That the dentists should unite and conduct an active campaign of education of the public in matters affecting the health of the mouth.

(3) That in order to accomplish great and lasting results, we must have the aid of the state and local boards of health and education.

I believe that the investigation by these four agencies should include the advisability of providing for proper and efficient methods of teaching the children of the schools the care and treatment of the mouth with frequent and persistent school inspection and toothbrush drills; that this will do more to insure oral cleanliness, prevent decay and act as a safeguard against the spreading of infection than any other method that has ever been proposed. It will help to establish in our schools the principles of personal hygiene.

There is certainly great need for a large amount of direct hygiene teaching in our schools, and it should include not only the teeth, but the body, eyes, skin and the digestion. All should be taught so carefully that every child will give answer in his habits and appearance.

I believe when the people, and especially the children, fully understand that tooth decay with its terrible consequences and excruciating pains is to a great extent preventable with clean mouths, that rapid progress with lasting and beneficial results will be attained.

The real issue of the question under consideration is whether mercenary profits received from the services of the hygienist will be commensurate with the loss to the profession for commercializing its aims and ideals. The merchant and the workman are primarily in business for the gains and profits they may receive. My ideal of the professional man would be shattered if I believed that his entrance into the professional field and the practice thereafter of his profession were guided and controlled by the desire to make money, and like a wolf in lamb's clothing use the cloak of a dignified profession to shield his actual practice as a mechanic and tradesman.

The true professional instinct is found in the man who places his calling on the high plane of devoting his education, his talents, his experience, his all, that humanity may be relieved of its sufferings, and that posterity may bless him for the legacy of good he has left them. To my way of thinking, it should be the desire of the dentist to make himself proficient even at a sacrifice of income.

—*The Dental Cosmos.*

